# Receptor Switching in Newly Evolved Adeno-associated Viruses

**DOI:** 10.1128/JVI.00587-21

**Published:** 2021-09-09

**Authors:** L. Patrick Havlik, Anshuman Das, Mario Mietzsch, Daniel K. Oh, Jonathan Ark, Robert McKenna, Mavis Agbandje-McKenna, Aravind Asokan

**Affiliations:** a Curriculum in Genetics and Molecular Biology, University of North Carolina at Chapel Hill, Chapel Hill, North Carolina, USA; b Department of Surgery, Duke University School of Medicine, Durham, North Carolina, USA; c Department of Biochemistry and Molecular Biology, Center for Structural Biology, McKnight Brain Institute, University of Florida, Gainesville, Florida, USA; d Department of Molecular Genetics and Microbiology, Duke University School of Medicine, Durham, North Carolina, USA; e Department of Biomedical Engineering, Duke University, Durham, North Carolina, USA; Cornell University

**Keywords:** CRISPR, cryo-EM, adeno-associated virus, capsid, gene therapy, receptor, structure-function

## Abstract

Adeno-associated viruses utilize different glycans and the AAV receptor (AAVR) for cellular attachment and entry. Directed evolution has yielded new AAV variants; however, structure-function correlates underlying their improved transduction are generally overlooked. Here, we report that infectious cycling of structurally diverse AAV surface loop libraries yields functionally distinct variants. Newly evolved variants show enhanced cellular binding, uptake, and transduction, but through distinct mechanisms. Using glycan-based and genome-wide CRISPR knockout screens, we discover that one AAV variant acquires the ability to recognize sulfated glycosaminoglycans, while another displays receptor switching from AAVR to integrin β1 (ITGB1). A previously evolved variant, AAVhum.8, preferentially utilizes the ITGB1 receptor over AAVR. Visualization of the AAVhum.8 capsid by cryoelectron microscopy at 2.49-Å resolution localizes the newly acquired integrin recognition motif adjacent to the AAVR footprint. These observations underscore the new finding that distinct AAV surface epitopes can be evolved to exploit different cellular receptors for enhanced transduction.

**IMPORTANCE** Understanding how viruses interact with host cells through cell surface receptors is central to discovery and development of antiviral therapeutics, vaccines, and gene transfer vectors. Here, we demonstrate that distinct epitopes on the surface of adeno-associated viruses can be evolved by infectious cycling to recognize different cell surface carbohydrates and glycoprotein receptors and solve the three-dimensional structure of one such newly evolved AAV capsid, which provides a roadmap for designing viruses with improved attributes for gene therapy applications.

## INTRODUCTION

Adeno-associated viruses (AAVs) are helper-dependent parvoviruses that have become a leading gene therapy vector, with Food and Drug Administration approvals in the United States and a growing list of clinical trials under way (1). While the majority of these indications use natural AAV isolates, attempts to improve the effectiveness of AAV vectors through capsid engineering are being pursued using a variety of methods, including rational design, DNA shuffling with natural serotypes, saturation mutagenesis of targeted regions, insertional mutagenesis, and error-prone PCR (2–4). These approaches have yielded new AAV capsid variants with different properties; however, the structure-function correlates of such are generally understudied, with only a few examples in the literature (5–8). Targeted modifications to improve transduction efficiency or alter tissue tropism will require a deeper understanding of AAV biology and correlating structural features of the capsid with specific functional attributes.

The AAV capsid is composed of 60 monomers of viral proteins (VPs) 1, 2, and 3, in a 1:1:10 ratio (9, 10). The sequences of VP1 to VP3 overlap, with the sequence of VP3 shared by all, VP2 N-terminally extended ∼66 amino acids relative to VP3, and VP1 N-terminally extended an additional ∼137 amino acids to VP2. The region unique to VP1 (VP1u) contains a phospholipase A2 domain (11), as well as a region responsible for interaction with GPR108 (12), which was recently identified as an essential host factor for a number of serotypes. The VP3 region forms the capsid surface, with a number of features common across AAVs (13, 14). These include a pore at the 5-fold axis of symmetry, essential for DNA packaging during assembly and externalization of VP1/2 N-terminal domains during trafficking; depressions at the 2-fold axis and surrounding the 5-fold pore; and protrusions at the 3-fold axis, often used to engage cell attachment factors, including glycans and the AAV receptor (AAVR). Glycan footprints at the 3-fold symmetry axis have been particularly well studied, with structures solved for AAV1/AAV6 and N-linked sialic acid (15), AAV2 and heparan sulfate proteoglycan (16–18), AAV4 and O-linked sialic acid (19, 20), AAV5 and N-linked sialic acid (21), and AAV9 and terminal N-linked galactose (22, 23). In addition, interactions of various AAVs and integrins (24–26), as well as AAV1/AAV2/AAV5 with AAVR, have also been mapped (27–30). Unlike the aforementioned AAV serotypes, the role of glycans or integrins in AAV8 transduction has not been determined, although the laminin receptor (31) and the recently discovered AAVR have been implicated (32). Further, liver transduction by AAV8 after systemic dosing has been shown to be enhanced by serum proteins such as albumin, transferrin, and low-density lipoprotein, although it is unclear whether such constitute receptor mediated uptake mechanisms (33). Other putative coreceptors for some AAVs have been reported, although no specific interactions or receptor footprints have been mapped to date (34–37).

While amino acid residues within the 3-fold symmetr*y* axis have been a common target for modification, exactly how evolution or engineering of different capsid surface loops results in improved transduction is not understood. Here, we utilize structure-guided evolution of the nonhuman primate isolate AAV8 to demonstrate that distinct capsid surface epitopes can in fact be evolved to impart different biological functions. Specifically, we identify the cell surface glycan and alternate receptor exploited by two distinct, newly evolved variants and map the structural footprints contributing to a newly evolved mechanism of cell entry, independent of the cognate AAV receptor. Our approach also underscores the potential to evolve AAV gene therapy vectors by precisely engineering different surface epitopes for novel receptor usage.

## RESULTS

### Evolution of distinct AAV surface epitope libraries yields improved variants.

**FIG 1:**
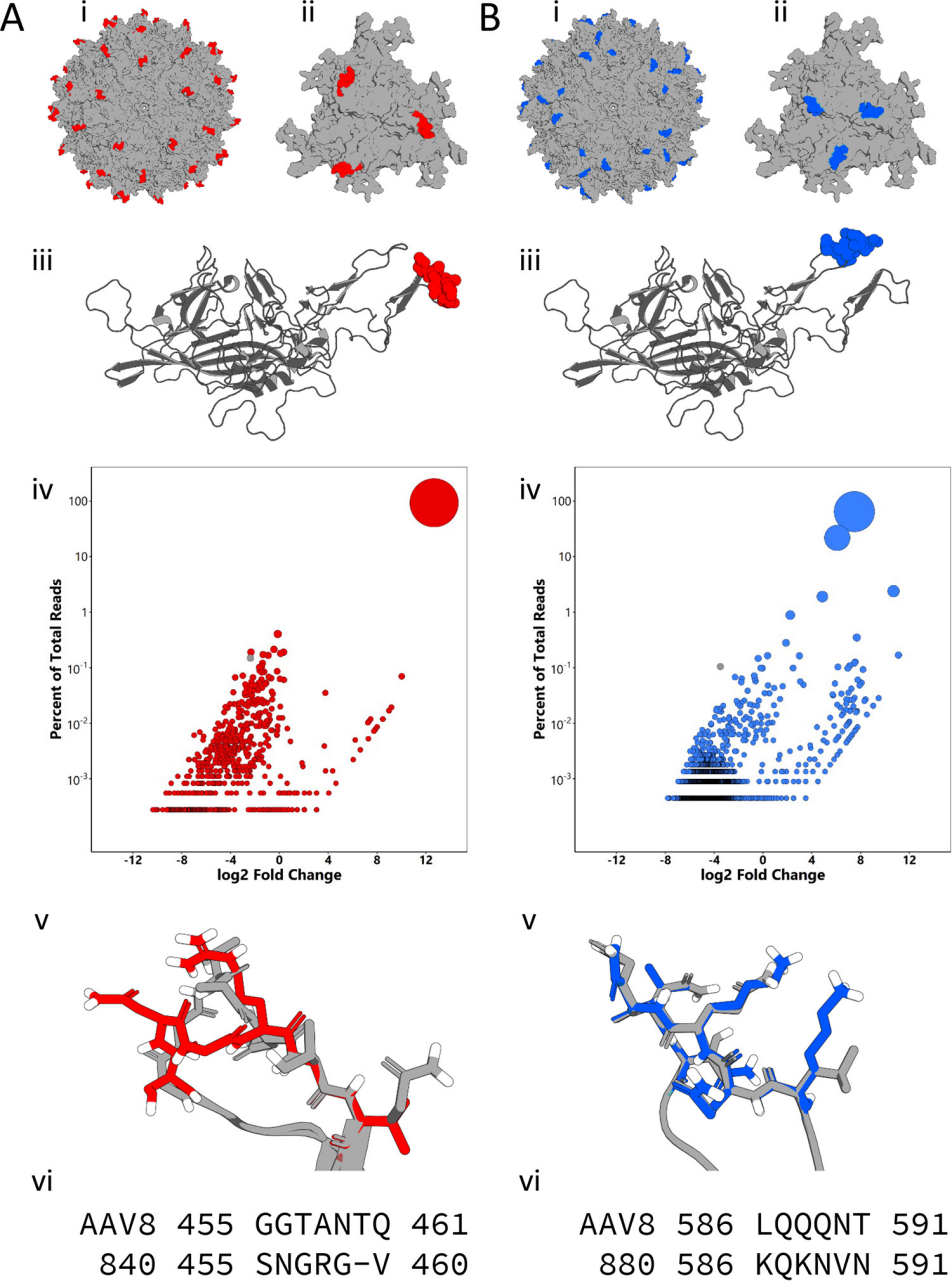
Evolution of distinct surface loops on the AAV capsid. Mutated variable regions IV (A) and VIII (B) are highlighted on full capsid (i), trimer (ii), and monomer (iii) models of AAV8 (PDB 2QA0). (iv) Bubble plots showing enrichment of capsid mutants after selection. Each bubble represents a unique amino acid sequence present after three rounds of selection. (v) Homology models of lead mutants were generated with SWISS-MODEL and COOT and aligned in PyMOL. WT AAV8 is shown in gray, the models of AAV840 are shown in red and those of AAV880 are shown in blue. (vi) Sequence alignments of the regions mutated and their respective lead mutants.

**FIG 2:**
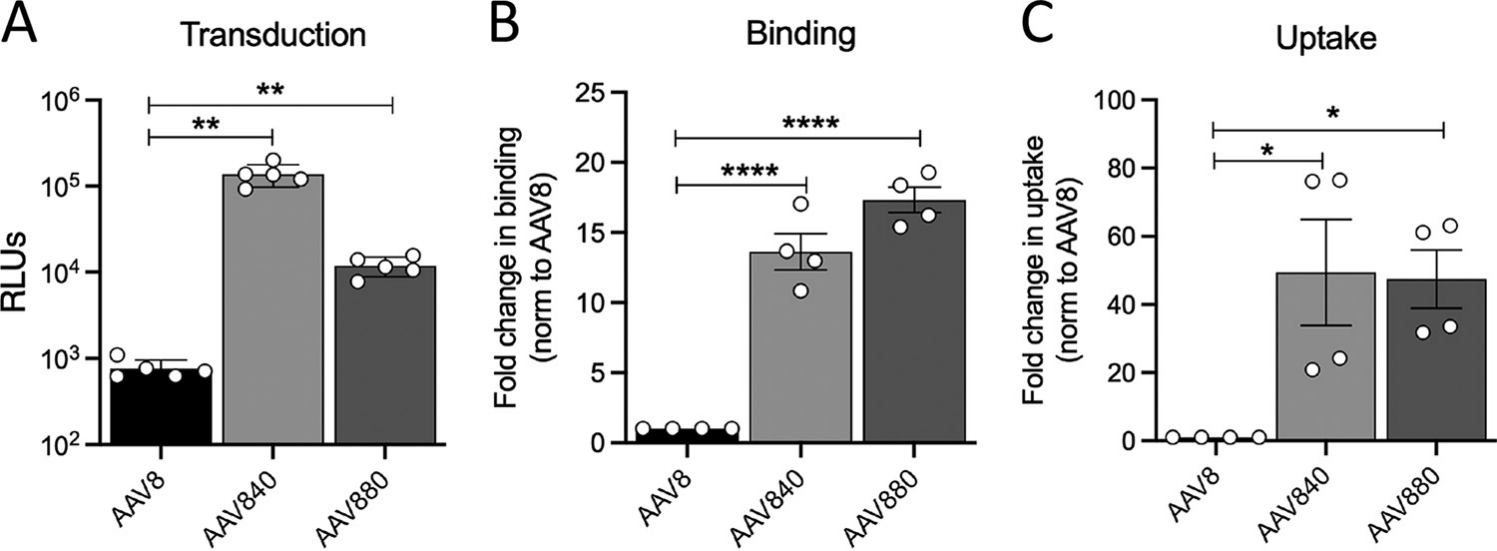
Cell surface binding, uptake, and transduction of mutant AAV capsids. (A) Mutant transduction of Huh7 cells using ssCBA-Luc vectors. (B and C) Cell surface binding (B) and virion internalization (C) on Huh7 cells. Vector genomes were measured by qPCR and expressed as the fold change in binding or uptake normalized to the parental AAV8 serotype. The data shown are from two to three technical replicates from two independent experiments. Error bars represent means ± the SD. Statistical significance (*P*) between groups was calculated by ordinary one-way ANOVA (*P < *0.05; **, *P < *0.01; ***, *P < *0.001; ****, *P < *0.0001).

The 3-fold axis of symmetry is important for multiple stages of parvovirus infection, including host cell attachment and postentry trafficking. In AAVs, glycan binding footprints from several serotypes are localized to this region, and anti-AAV neutralizing antibodies (NAbs) commonly target these footprints. In order to further explore the relationship between structure and the potential functions of this part of the capsid, we created saturation mutagenesis libraries on two distinct surface loops localized to the spikes protruding from the 3-fold axis at variable regions (VRs) IV and VIII (Fig. 1A and B, i to iii). Infectious cycling of these libraries was carried out with each round of selection involving infection of human hepatoma Huh7 cells with each AAV library, followed by superinfection with human adenovirus 5 (Ad5). After multiple rounds of cycling, the AAV libraries were sequenced on the MiSeq high-throughput sequencing platform, and the results were analyzed with a custom Perl script. Each library yielded one mutant that accounted for a majority of the MiSeq reads (Fig. 1A and B, iv), dubbed AAV840 and AAV880 for the VR-IV and VR-VIII mutants, respectively. The evolved AAV840 sequence has one fewer amino acid than the region targeted for the library, changing from 455-GGTANTQ-461 to 455-SNGRGV-460. It was noted that the evolved AAV840 sequence also contains an NGR motif, previously shown to mediate AAV-integrin interactions (25). The sequence for AAV880 changed from 586-LQQQNT-591 in AAV8 to 586-KQKNVN-591. The two positively charged lysines in close proximity are reminiscent of two similarly clustered arginines, important for heparan sulfate proteoglycan binding, in the same VR8 region of AAV2 (17). Both AAV variants demonstrated increased transduction in human hepatoma cells *in vitro* by 1 to 2 orders of magnitude over the parental AAV8 capsid (Fig. 2A). This was mediated at least in part by significant increases in cellular binding and internalization for both variants (Fig. 2B and C). Taken together, these data demonstrate that evolution of variable regions of the AAV8 capsid significantly improves transduction efficiency *in vitro*. Although not statistically significant, it is interesting to note that despite the slightly lower cell surface attachment compared to AAV880, the AAV840 variant displayed efficient uptake and improved transduction efficiency.

### Newly evolved AAV variants exploit distinct glycans for cell surface attachment.

**FIG 3:**
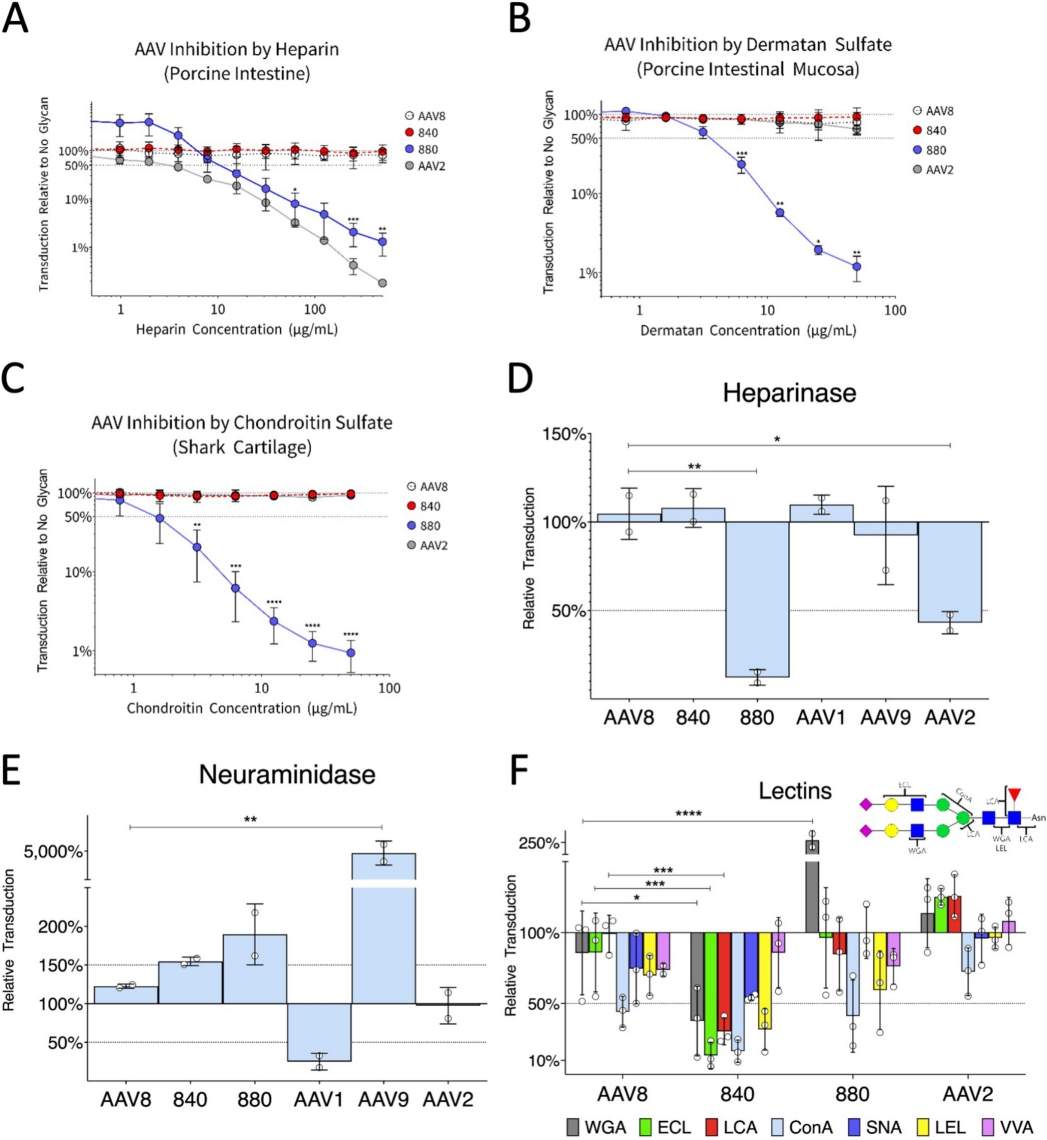
Glycan preferences of different AAV variants. (A to -C) Capsids were incubated with increasing concentrations of various sulfated glycosaminoglycans for 30 min at room temperature before transduction of Huh7 cells. (D to E) Cells were treated with heparinase or neuraminidase to remove surface heparin or sialic acid, respectively, before transduction with AAV vectors. (F) Cells were preincubated with various lectins at 4°C for 30 min, followed by the addition of AAV vector and incubation for an additional 30 min. Unbound lectin and vector was washed away, and cells were returned to the incubator. The inset shows where each lectin that inhibits 840 binds on a typical N-linked glycan. Full lectin names are listed in Materials and Methods. Data in all panels are normalized to untreated controls and represent means ± the SD. The horizontal dotted lines represent 50% inhibition of AAV transduction. Data shown are from two to three independent experiments with two technical replicates each. Error bars represent means ± the SD. Statistical significance (*P value*) between groups was calculated by an ordinary one-way ANOVA (*P < *0.05; **, *P < *0.01; ***, *P < *0.001; ****, *P < *0.0001).

In order to further understand the mechanism of increased transduction of the evolved mutants, we investigated their dependence on common attachment factors. Preincubation of virus with soluble heparin significantly inhibits serotypes that are dependent on heparan sulfate proteoglycans (HSPG) for efficient cellular attachment, such as AAV2 (38). We first observed that the AAV880 variant was inhibited by soluble heparin in a concentration-dependent manner while AAV8 and AAV840 were unaffected (Fig. 3A). Similarly, pretreatment of cells with heparinase to remove surface HSPG almost completely blocked AAV880 transduction while having no effect on AAV8 or AAV840 (Fig. 3D). Interestingly, AAV880 was also very strongly inhibited by other sulfated glycosaminoglycans that do not block AAV2 transduction. Namely, soluble chondroitin sulfate and the closely related dermatan sulfate specifically inhibited AAV880, whereas AAV2, AAV8, and AAV840 were all unaffected (Fig. 3B and C). Neuraminidase pretreatment of cells serves the dual purpose of blocking the transduction of serotypes that utilize sialic acid for attachment, such as AAV1, as well as increasing transduction of serotypes such as AAV9 that utilize terminal N-linked galactose, which precedes sialic acid on a typical N-linked glycan (22). However, in neuraminidase-pretreated cells, no significant difference was observed in the relative transduction of AAV840 and AAV880 compared to AAV8 (Fig. 3E), indicating they exploit neither sialylated nor galactosylated glycans for attachment. Finally, virus attachment can be competitively inhibited by preincubating cells with lectins that bind the same glycan as the virus. We found that a number of lectins were able to block transduction by AAV840 (Fig. 3F). However, these lectins do not share specificity for a single glycan; rather, they bind all along a typical N-linked glycan, possibly inhibiting AAV cell surface attachment in a nonspecific manner. Thus, evolving VR-VIII on the capsid surface yielded a novel AAV variant that recognizes sulfated glycosaminoglycans, while the evolved VR-IV appears to prefer no cell surface glycan in particular. This raised the possibility that the increased transduction demonstrated by AAV840 is mediated by a protein, not attachment to a particular glycan. Rather than preventing AAV840-glycan interactions, the inhibitory lectins may bind glycans decorating a protein host factor, thereby sterically hindering access to the AAV840 capsid.

### A CRISPR/Cas9 genome-wide screen reveals ITGB1 as an essential host factor for selected AAV variants.

**FIG 4:**
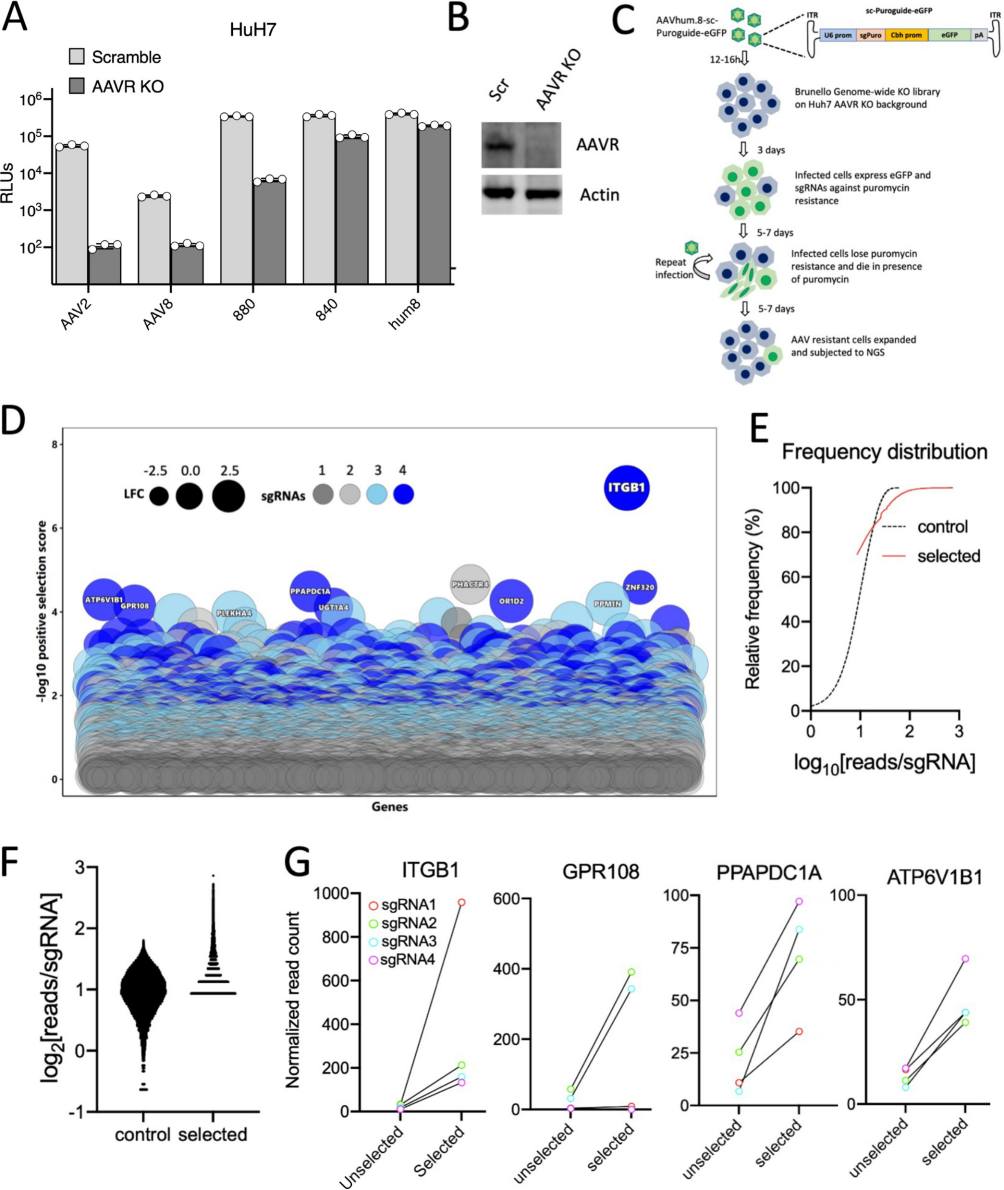
CRISPR/Cas9 genome-wide screen identifies essential host factors for AAVhum.8. (A) Transduction of Huh7 Scramble and *AAVR* knockout cells with AAV8 and its evolved variants packaging recombinant ssCBA-Luc genomes. (B) Western blot of Huh7 Scr and AAVR KO cells (C) Schematic of a genome-wide CRISPR/Cas9 survival screen for AAVhum.8 essential host factors. (D) Bubble plot of genes identified by NGS of sgRNAs from surviving cells. (E) Relative frequency distribution of sgRNAs in control and selected samples in the screen. (F) Log_2_ number of reads per sgRNA sequenced in the control and selected populations. (G) Normalized sgRNA read count of *ITGB1* and other top hits in the screen. Data shown in panel A are from a representative experiment with three technical replicates for each group. Error bars represent means ± the SD.

A number of entry receptors have been identified for various AAVs, including AAVR, which is essential for efficient transduction by most AAVs (32). However, AAV4 and Rh32.33 are completely independent of AAVR, while most AAVs exhibit markedly decreased transduction in AAVR knockout cells (39). To determine whether our newly evolved AAV variants were dependent on AAVR, we first transduced Huh7 AAVR knockout (KO) cells alongside parental AAV8 and AAV2 serving as positive controls (Fig. 4A and B). The AAV8 variants not only showed significantly higher transduction (ca. 1 to 3 logs) but also showed greater independence from AAVR compared to the parental AAV8 (Fig. 4A), implying a potential switch in receptor preference. Importantly, while the AAV880 variant remains largely dependent on AAVR, the AAV840 and its evolved variant AAVhum.8, which carries additional mutations to aid with NAb evasion, showed nearly complete independence from AAVR, implying a receptor switching event.

**FIG 5:**
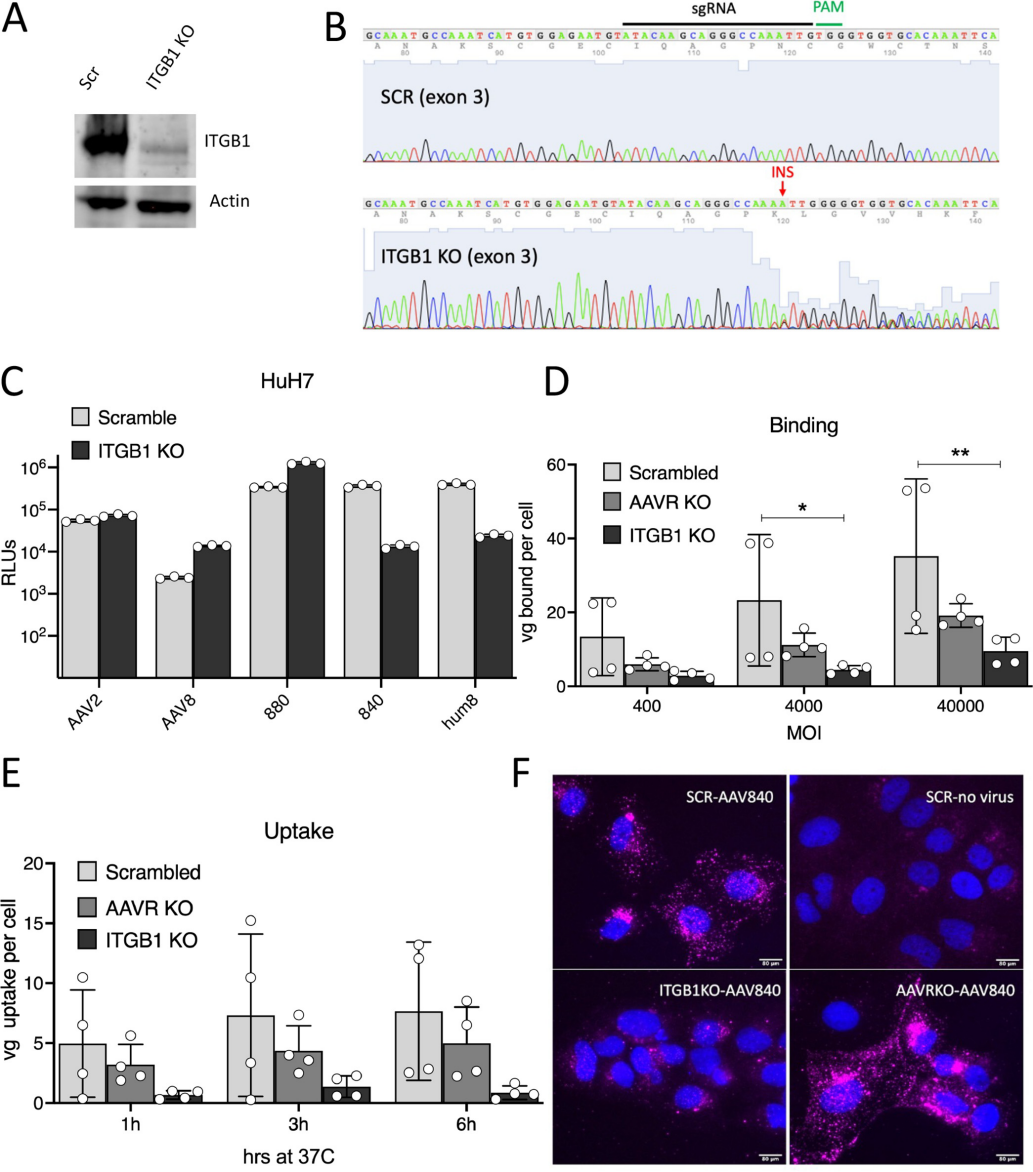
ITGB1 is an essential receptor for binding and uptake of AAVhum.8. (A) Western blot of Huh7 Scr and ITGB1 KO cells. (B) Sanger sequencing of sgRNA targeting region in exon 3 of *ITGB1* gene shows single base insertion in the *ITGB1* KO cells, resulting in a frameshift. (C) Transduction of Huh7 Scramble and *ITGB1* knockout cells with AAV8 and its evolved variants packaging recombinant ssCBA-Luc genomes. (D and E) Cell surface binding (D) and uptake (E) of AAVhum.8 on Huh7 Scramble, *AAVR* KO, and *ITGB1* KO cells. (F) Immunofluorescent images showing AAV840 intact capsid (magenta) internalized 6 h after virus addition at 37°C. Nuclei were counterstained with Hoechst 33342. Images were acquired at ×10 magnification. Data shown in panel C are from a representative experiment, with two technical replicates each. Data shown in panels D and E are from two independent experiments, with two technical replicates each. Error bars represent means ± the SD. Statistical significance (*P*) between groups was calculated by a two-way ANOVA (*P < *0.05; **, *P < *0.01; ***, *P < *0.001; ****, *P < *0.0001).

To understand which host factors might be responsible for the improved and AAVR-independent transduction displayed by AAV840 and AAVhum.8, we conducted a high-throughput, genome-wide screen using the Brunello CRISPR/Cas9 knockout library (40) on Huh7 AAVR KO cells. Briefly, cells in the library were transduced with an AAVhum.8 vector expressing green fluorescent protein (GFP), as well as two guide RNAs targeting the puromycin resistance gene integrated into the genome during creation of the library (Fig. 4C). Successfully transduced cells died under puromycin selection leaving behind survivor cells resistant to AAVhum.8 transduction. After two rounds of puromycin selection and expansion, genomic DNA from the survivor cell population was isolated and subjected to next-generation sequencing (NGS) to determine the single-guide RNAs (sgRNAs) enriched in comparison to an uninfected control library (Fig. 4C). A subset of sgRNAs were highly enriched in both frequency and absolute read counts in the selected population compared to the control, highlighting the robust positive selection obtained in our screen (Fig. 4E and F). Further, sgRNA ranking analysis revealed at least seven genes that had all four sgRNAs enriched with the lowest *P* values (*P ≤ *2.98 × 10^−4^) (Fig. 4C). Among them, sgRNAs targeting *ITGB1*, encoding the integrin β1 subunit, were the most enriched (FDR ≤ 0.01, *P ≤ *2.59 × 10^−7^) and emerged as the top hit of the screen (Fig. 4D). Interestingly, along with *ITGB1*, *GPR108*, a known entry factor for AAV (12), appeared among the top 10 hits (Fig. 4G), further validating our screen. To independently validate the role of *ITGB1* in transduction of AAVhum.8 and other AAV8 variants, we used CRISPR/Cas9 editing to create an *ITGB1* knockout on the Huh7 cell background using a previously validated sgRNA targeting the exon 3 of the gene (41). Protein expression analysis, as well as sequencing of the targeted exon region, confirmed knockout of the integrin β1 protein subunit (Fig. 5A and B). Next, we transduced AAVhum.8, AAV840, and AAV880 variants on *ITGB1* KO cells and observed significantly reduced transduction by AAVhum.8 and its predecessor AAV840 (∼1.5 log) compared to scrambled cells (Fig. 5C). In contrast, AAV880 variant, parental AAV8, and AAV2 transduction remained at wild-type levels or higher on *ITGB1* KO cells (Fig. 5C), indicating a divergence in receptor preference of AAVhum.8 and AAV840 variants, resulting in a switch from AAVR- to ITGB1-mediated transduction.

To further understand whether ITGB1 mediates early entry steps, we performed binding assays by incubating AAVhum.8 on cells at 4°C and determined the amount of vector genomes bound by qPCR. Compared to control cells, at least 10-fold less virus was bound to *ITGB1* KO cells, whereas only ca. 2- to 3-fold less virus was bound to *AAVR* KO cells (Fig. 5D), indicating that AAVhum.8 binding on Huh7 is highly dependent on ITGB1 but not AAVR. Next, we measured the uptake of AAVhum.8 virions by incubating synchronously infected cells at 37°C and quantifying internalized vector genomes at various time intervals using qPCR. We found that uptake of AAVhum.8 virions was greatly reduced in *ITGB1* KO cells (>10-fold) compared to *AAVR* KO (<2-fold) cells (Fig. 5E). To visualize the influence of ITGB1 on the cellular uptake of AAV capsids, we infected cells with AAV840 (the precursor variant, since no available anti-AAV8 antibodies recognize AAVhum.8) and performed immunofluorescent staining with an AAV8 monoclonal antibody, ADK8. We observed that a significant number of intact AAV840 capsids were internalized 6 h postinfection in Huh7 scrambled and *AAVR* KO cells but not in *ITGB1* KO cells (Fig. 5F). Taken together, these data highlight receptor switching from AAVR to ITGB1, while evolving variable region IV (VR-IV), but not VR-VIII AAV8 variants.

### Cryo-EM visualization and mutational analysis of AAV variants reveals structure-function correlates of ITGB1 receptor usage.

**FIG 6:**
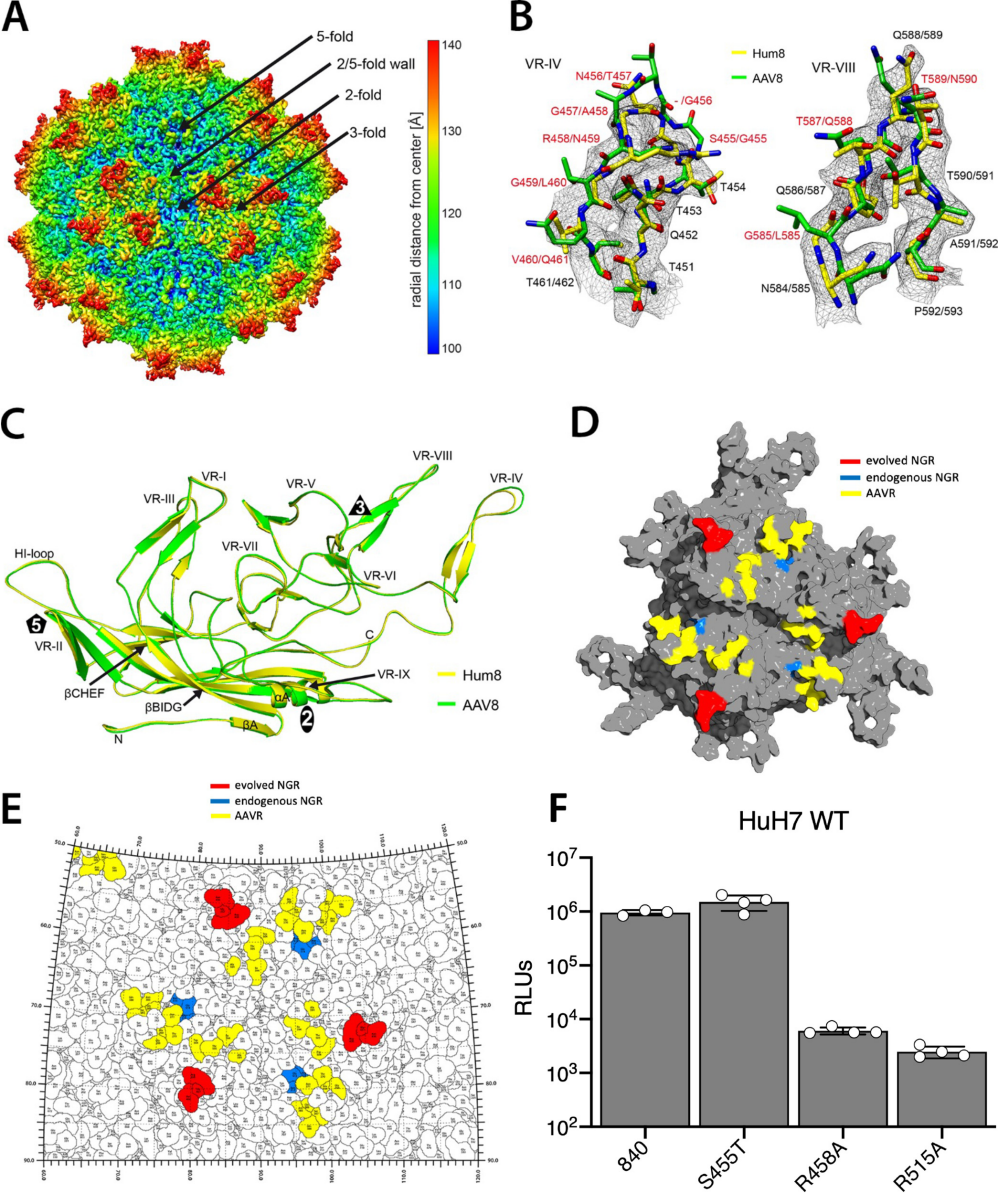
Determination of AAVhum.8 capsid structure and structure-function analysis. (A) Reconstructed AAVhum.8 capsid map colored according to radial distance from the capsid center (blue) to outermost regions (red), as indicated by the scale bar on the right. The positions of the 5-fold channel, 3-fold, 2-fold axes, and 2/5-fold wall are indicated. (B) The modeled AAVhum.8 VR-IV and VR-VIII surface loops are shown in stick representation inside its mesh density map. AAV8 (PDB-ID 6V12) shown in green is superposed onto AAVhum.8 (yellow). The amino acids are as labeled. The amino acid substitutions of AAVhum.8 relative to AAV8 are displayed in red. (C) Structural superposition of AAV8 (green) and AAVhum.8 (yellow) shown as ribbon diagrams. The positions of the N and C termini, the core β-sheets, the α-helix, and the variable regions (VRs) are indicated. This figure was generated using PyMOL (62). (D) AAVhum.8 capsid VP trimer with colored surface footprints for the AAVR (yellow), endogenous integrin recognition domain (blue) and evolved integrin recognition domain (red). (E) Roadmap projection of surface residues within different receptor footprints on the AAVhum.8 3-fold symmetr*y* axis. (F) Mutational analysis of the putatively evolved versus extant integrin recognition NGR motifs in AAV840 and impact on transduction efficiency *in vitro*. The data shown are from a representative experiment, with three technical replicates for each group. Error bars represent means ± the SD.

To further characterize the AAVhum.8 variant its capsid structure was determined by cryo-EM. Utilizing 275,883 individual particle images the AAVhum.8 capsid was reconstructed to 2.49 Å resolution (Table 1). The AAVhum.8 density map displayed the characteristic morphological features of other AAVs, e.g., a channel at the 5-fold symmetr*y* axes, trimeric protrusions that surround each 3-fold symmetr*y* axis, a depression at each 2-fold symmetr*y* axis and shows high similarity to the previously described AAV8 capsid (42) (Fig. 6A). In the high-resolution density map, amino acid side chains were clearly visible, and a model was built for AAVhum.8 based on the AAV8 capsid structure (PDB 6V12). Compared to AAV8, the capsid structure of AAVhum.8 is highly conserved except for VR-IV that displays major structural variation with a Cα distance of up to 3.2 Å (Fig. 6B). This difference is caused by the evolved sequence in VR-IV, including a single amino acid deletion that results in a slightly shorter loop relative to AAV8 (Fig. 6C). Notably, the newly evolved VR-IV residues comprise of a highly conserved putative integrin recognition motif NGR, also located endogenously on most AAV serotypes. The remaining amino acid substitutions in VR-V, VR-VIII, and the HI-loop compared to AAV8 are visible in the density map but do not lead to alternative surface loop conformations. The overall Cα-RMSD for the entire AAVhum.8 VP when superposed to AAV8 is 0.52 Å, which is comparable to other closely related clade E capsids such as AAVrh.10 or AAVrh.39 (42). Additional structural models highlighting the AAVR footprint, as well as the newly evolved and endogenous integrin binding motifs (Fig. 6D and E), were then analyzed. These surface and roadmap renditions of the 3-fold symmetr*y* axis further corroborated that the VR-IV integrin motif lies adjacent to the AAVR footprint residues without overlap.

**TABLE 1:** Summary of data collection and image-processing parameters

Parameter	AAVhum.8
Data collection statistics	
Total no. of micrographs	1,789
Defocus range (μm)	0.56–2.00
Total electron dose (e^–^/Å^2^)	60
Frames/micrograph	75
Pixel size (Å/pixel)	1.10
No. of capsids used for final map	275,883
Resolution of final map (Å)	2.49
	
Refinement statistics	
Map CC	0.888
RMSD bonds (Å)	0.01
RMSD angles (°)	0.92
All-atom clashscore	3.91
	
Ramachandran plot (%)	
Outliers	0
Allowed	2.5
Favored	97.5
Rotamer outliers	0
C-beta deviations	0

To assess the relative contributions of the putative newly evolved versus extant integrin recognition motifs in AAV840 (i.e., 456-NGR-458 versus 513-NGR-515 residues), we generated Arg-to-Ala mutants within the respective surface epitopes. As seen in Fig. 6F, both the R458A mutation in the evolved NGR motif and the R515A mutation in the endogenous motif profoundly impacted transduction efficiency, while a minor S455T mutation in the evolved sequence but adjacent to the NGR had no effect. These results further corroborate that the newly evolved VR-IV loop in AAV840 and AAVhum.8 is essential to trigger the receptor switch to integrin β1. Further, these observations also underscore the ability to evolve nonredundant receptor preferences in AAV capsids through mutagenesis and engineering of structurally distinct surface domains.

## DISCUSSION

Mapping of structure-function correlates for natural AAVs has greatly improved our understanding of their infectious pathways and tissue tropism. Simultaneously, this knowledge has also informed the design of AAV variants with novel, desirable properties, whether through rational design or directed evolution. Despite these advances, the mechanisms by which newly evolved AAV variants often show improved transduction profiles or altered tropism remain poorly understood. In this study, we set out to understand the functional consequences of randomizing and evolving distinct structural loops on the AAV capsid surface. To date, random peptide insertion has largely relied on the VR-VIII surface loop, regardless of the parental serotype (43–46). This approach hinges on the simple assumption that novel function is often imparted to the AAV capsid due to the linear sequence of the peptide insert. Residues flanking the peptide insert often play a role in efficient surface presentation. However, for naturally occurring surface epitopes, the AAV capsid presents a three-dimensional (3D) structure with residues constituting potential receptor binding footprints often spread across the primary amino acid sequence of the VP3 protein subunit but arranged within the quaternary structure of an assembled virion (15, 16, 18, 27). Thus, while being subject to evolutionary pressure, mutations that impart novel function but that also minimally disrupt AAV capsid assembly are likely to be preferred. Further, we have previously demonstrated that coevolution of antigenicity and tropism in overlapping AAV capsid surface footprints is feasible; for instance, AAVhum.8 effectively evaded anti-AAV8 neutralizing antibodies, while simultaneously demonstrating expanded tropism for human hepatocytes in a murine liver xenograft model (47).

Here, we independently evolve distinct variable regions, VR-IV and VR-VIII, located on opposite ends of the 3-fold spikes on the AAV capsid surface. Although both approaches yielded improved AAV variants, we observed that they do so using distinct mechanisms. The AAV880 variant, which carries newly acquired residues in VR-VIII, is reminiscent of both the sequence and the function of the same region in AAV2. While AAV2 contains two arginines in close proximity and specifically binds heparan sulfate proteoglycan, AAV880 has two clustered lysines in a similar position and interacts with sulfated glycosaminoglycans. However, AAV880 notably appears to have stronger preference for chondroitin sulfate over heparan sulfate. This convergence of phenotype in VR-VIII may imply that this loop is predisposed to recognition of cell surface glycans, particularly sulfated glycosaminoglycans that are abundantly expressed. For instance, AAV8 has previously been shown to bind heparan sulfate with a single E533K mutation (48), a position that is brought in close proximity to the two lysines of AAV880 upon interdigitation of VP monomers.

In contrast, the AAV840 variant, which carries newly acquired residues in VR-IV, did not display any glycan preference, instead demonstrating a markedly decreased dependence on the cognate AAV receptor (AAVR). This observation is even more striking in the case of AAVhum.8, which was evolved from the predecessor AAV840 variant and carries additional mutations to aid with NAb evasion (47). This striking independence from AAVR prompted us to carry out an essential host factor screen using AAVhum.8 and the Brunello CRISPR genome-wide knockout library on an AAVR-null (*AAVR* KO) background. The top hit in the screen *ITGB1*, encoding integrin subunit β1, has been previously implicated in AAV entry for certain cell types (24–26). However, what is particularly striking with this finding is the specificity of AAV840 and AAVhum.8 toward ITGB1. Although AAV2 and AAV8, as well as the VR-VIII variant, AAV880, showed no dependence on ITGB1, AAVhum.8 and the precursor VR-IV variant, AAV840, significantly rely on ITGB1 for improved transduction. When taken together with the acquired AAVR independence displayed by AAV840/hum.8 over AAV2/8/880 capsids, this important finding suggests that the newly evolved integrin recognition motif displays a structural context dependent phenotype, enabling a switch in receptor usage. Mechanistically, this novel receptor interaction appears to facilitate improved cell surface binding and internalization compared to AAVR-mediated transduction.

The cryoelectron microscopy (cryo-EM) structure of the AAVhum.8 variant further corroborates these functional observations. First, we accurately map the evolved integrin recognition footprint responsible for the observed receptor switching event. Interestingly, the newly evolved VR-IV residues are positioned adjacent to the disrupted AAVR footprint residues on the AAVhum.8 capsid surface. However, it is critical to note that the AAVR residues are not mutated in the precursor AAV840 variant, implying that the newly acquired VR-IV NGR motif alone is sufficient to enable the switch to ITGB1 from AAVR. Taken together, our results highlight the important finding that the AAV capsid likely evolved distinct and redundant strategies to exploit distinct surface glycans and receptors that facilitate cell attachment and entry. We provide evidence for such through forward evolution, demonstrating that distinct surface loops on the AAV capsid can be evolved to impart distinct biological properties. While outside the scope of this discussion, it is important to note that the capsid-host interactions discovered in the present study will require further investigation *in vivo*, while taking into account the impact of serum factors and viral uptake by non-hepatocyte cell populations. Our findings also provide a roadmap for tailoring distinct receptor preferences independent of the cognate AAV receptor, which might enable gene transfer at a higher efficiency compared to natural AAV serotypes.

## MATERIALS AND METHODS

### Cell culture and viruses.

HEK293 and Huh7 cells were cultured in Dulbecco modified Eagle medium (DMEM; Gibco, catalog no. 11995065) supplemented with 10 and 5% fetal bovine serum (FBS), respectively, and 100 U/ml penicillin/10 μg/ml streptomycin (Gibco, catalog no. 15140148) in 5% CO_2_ at 37°C. Human adenovirus 5 (Ad5) was purchased from the American Type Culture Collection (ATCC VR-1516). AAV840 integrin mutants were created by site-directed mutagenesis using Q5 Hot Start Polymerase (NEB, M0493S).

### AAV vector production, purification, and quantification.

Recombinant AAV vectors were produced by transfecting HEK293 cells at 70 to 80% confluence with polyethylenimine (PolySciences, catalog no. 24765-1) using triple-plasmid transfection (49). Recombinant vectors packaging single-stranded genomes encoding firefly luciferase driven by the chicken β-actin promoter (ssCBA-Luc) or a hum.8 vector packaging a self-complementary genome expressing two puromycin-resistance-targeting-sgRNAs driven by the U6 promoter and a fluorescent marker (eGFP) downstream, driven by a human EF1-alpha core promoter (AAVhum.8 sc-Puroguide-eGFP) were generated using this method. AAV libraries were produced by double transfection of pTR-AAV8-Library plasmid and adenovirus helper pXX680. Subsequent steps involving the harvesting of recombinant AAVs and downstream purification were carried out as described earlier (49). Recombinant AAV titers were determined by quantitative PCR with primers amplifying the AAV2 inverted terminal repeat regions (ITRs) 5′-AACATGCTACGCAGAGAGGGAGTGG-3′ and 5′-CATGAGACAAGGAACCCCTAGTGATGGAG-3′.

### Generation of AAV capsid libraries.

AAV capsid libraries were engineered through saturation mutagenesis of variable loop regions identified using cryo-EM as previously described (47). Briefly, a library insert containing degenerate nucleotides at the selected region was assembled from overlapping single-stranded oligonucleotides with Gibson Assembly Master Mix (NEB, E2611S) and ligated between the BsiWI and SbfI sites in pTR-AAV8**. The pTR-AAV8** plasmid contains AAV2 ITRs flanking AAV2 Rep and AAV8 Cap. Importantly, amino acids 446 and 447 in AAV8 Cap were mutated to stop codons in order to reduce WT AAV8 plasmid contamination.

### Infectious cycling of AAV libraries.

Huh7 cells at ∼75% confluence were infected overnight with AAV8 libraries at 5,000 viral genomes per cell. Media was then replaced with fresh media containing Ad5 at a multiplicity of infection (MOI) of 0.5. The supernatant medium was collected at 50 to 75% cytopathic effect, incubated at 55°C for 30 min to inactivate the Ad5, and centrifuged to remove cell debris. In total, three cycles of selection were completed before subjecting the evolved library to NGS. At each round of infectious cycling, AAV8 variants secreted into the supernatant were quantified by quantitative PCR using AAV2 ITR primers.

### Identification of newly evolved AAV mutants.

To analyze the sequence diversity of the parental and evolved AAV libraries, DNase I-resistant viral genomes were isolated from media and amplified by Q5 Hot Start Polymerase (NEB, M0493S) for 10 to 18 cycles using the primers 5′-CCCTACACGACGCTCTTCCGATCTNNNNNGTACCTGTACTACTTGTCTCG-3′ and 5′-GACTGGAGTTCAGACGTGTGCTCTTCCGATCTNNNNNAGACCATACCGGGTAAG-3′ and then sequenced with the MiSeq platform as previously described (47).

### Sequencing data analysis.

Demultiplexed reads were analyzed with an in-house Perl script (47). In brief, reads were searched for the nucleotide sequences flanking each library region, and the occurrence of each nucleotide sequence between these flanking motifs was counted and ranked. The sequences were also translated, the resulting amino acid sequences were counted, and their percent representations were calculated and ranked. Parental and evolved libraries were compared to determine the enrichment of selected variants, and sequences were plotted according to their enrichment and representation in each library using the R graphics package v4.0.2.

### Transduction and competitive inhibition assays.

For transduction assays, ssCBA-Luc vectors were added to cells in 24-well (50,000 cells/well) or 96-well plates (1e4 cells/well) at 10,000 vg/cell and incubated at 37°C for 48 h. Cells were then lysed in 1× passive lysis buffer (Promega, E1941) for 30 min at room temperature. Luciferase activity was measured with a Victor X3 plate reader (Perkin-Elmer) immediately after the addition of 25 μl of luciferin (Promega, E1483) to 25 μl of cell lysate. For competitive inhibition with soluble glycans, ssCBA-Luc vectors were combined with the appropriate glycan in black, clear-bottom 96-well plates (Corning, catalog no. 3904) in serum-free DMEM, followed by incubation at room temperature for 30 min. A total of 10,000 cells were then added to each well in 50 μl of DMEM–10% FBS, followed by incubation at 37°C for 48 h before processing as described above. For enzymatic removal of surface glycans, cells were incubated in serum-free DMEM containing 0.05 U/ml neuraminidase (Sigma, N7885) or 0.1 U/ml heparinase (Sigma, H2519) for 2 to 3 h at 37°C and then chilled at 4°C for 30 min. Virus was added, and incubation continued at 4°C for 1 h. The medium was then removed, and the cells were washed with phosphate-buffered saline (PBS) to remove unbound virus before returning to 37°C for 48 h and processing as described above. For lectin experiments, cells were chilled to 4°C for 30 min before replacing the medium with SF-DMEM containing 100 μg/ml of the appropriate lectin (Vector Labs) and continuing incubation for 30 min. Virus was then added, followed by incubation for a further 30 min at 4°C. The medium/lectin/virus was removed, and the cells were washed with PBS and returned to the incubator for 48 h before being processed as described above. The lectin abbreviations were as follows: WGA, wheat germ agglutinin; ECL, *Erythrina cristagalli* lectin; LCA, *Lens culinaris* agglutinin; ConA, concanavalin A; SNA, Sambucus nigra lectin; Lycopersicon esculentum lectin; and VVA, *Vicia villosa* lectin.

### Viral binding and uptake assays.

Viral binding assays were performed on Huh7 scrambled, ITGB1 KO, and AAVR KO cells. Briefly, 2.5 × 10^5^ cells were plated in duplicate in each well of a 24-well plate. Next day, various dilutions of AAVhum.8 virus inoculum, starting with ∼40,000 vector genomes (vg)/cell (∼2 × 10^10^ vg), which was diluted 10-fold twice to obtain 4,000 vg/cell and 400 vg/cell, were incubated at 4°C for 1 h. To remove unbound virus, the cells were washed three times with cold PBS and lysed directly in 100 μl of viral lysis buffer. Viral uptake assays were performed as described above except only 40,000 vg/cell inoculum was used and cells were shifted from 4 to 37°C, followed by incubation for 1, 3, and 6 h. Cells were washed three times with PBS and trypsinized to remove surface-bound virus. Cells were pelleted at 500 × *g* for 3 min and lysed in 100 μl of viral lysis buffer. Lysates were further processed for real-time PCR using AAV ITR primers as described previously (50). An inoculum of 100,000 vg/cell was used in assays comparing AAV8 and evolved variants (Fig. 2B and C) due to the low binding and uptake of AAV8 on Huh7 cells.

### Immunofluorescent staining of AAV840 variant.

In an 8-well chamber glass slide, ∼50,000 Huh7 scrambled, *ITGB1* and *AAVR* KO cells were plated and incubated overnight at 37°C. Next day, cells were incubated with AAV840 (∼6.5 × 10^9^ vg/well or 650,00 vg/cell) at 4°C for 1 h, washed three times with PBS, and moved to 37°C for another 6 h. Cells were washed twice with PBS and fixed in 4% paraformaldehyde for 20 min. Permeabilization and blocking were done in PBS containing 0.1% saponin and 1% bovine serum albumin for 1 h, followed by incubation with ADK8 antibody (1:50 dilution) in the same buffer overnight at 4°C. Primary antibody was removed by three washes with PBS containing 0.05% saponin, followed by incubation with secondary goat anti-mouse 647 antibody (Abcam, catalog no. ab150115) for 1 h at room temperature. Excess antibodies washed with PBS and nuclei were counterstained with Hoechst 33342 (Invitrogen, H3570) diluted 1:2,000 in PBS for 15mins. Each slide was mounted with Prolong Gold antifade mountant (Invitrogen, P36930) and imaged under a 10× Olympus objective fitted in an ECHO Revolve fluorescence microscope. Images were exported, and a scale bar was added using ImageJ.

### Genome-wide CRISPR/Cas9-based screen for essential AAVhum.8 host factors.

A human genome-wide Brunello plasmid library containing ∼80,000 sgRNAs targeting ∼20,000 human genes (Addgene, catalog no. 73179) was a gift from Stanley Lemon (UNC-Chapel Hill). To generate the lentivirus library, 10 μg of Brunello pDNA pool, 7.5 μg of psPAX2 (Addgene, catalog no. 12260), and 3 μg of pVSVG (Addgene, catalog no. 8454) were resuspended in 500 μl of serum-free DMEM and mixed with 500 μl of serum-free DMEM containing PEI-max (5 μl/μg of DNA), followed by incubation at room temperature for 15 min. The solution was added dropwise on 1 × 10^7^ HEK293 cells seeded on a 15-cm dish. Lentivirus concentration and titration were done as described previously (51).

Huh7 AAVR KO cells were transduced with an appropriate volume of the lentivirus library to obtain a coverage of ∼200×, selected with 3 μg/ml puromycin for a week, and used directly for the screen. The screen was initiated by plating ∼1 × 10^7^ cells from the library in each 15-cm dish (∼125× coverage), and two such dishes representing ∼250× coverage were considered one technical replicate. The screen was conducted with two technical replicates with each dish infected with 5 × 10^11^ vg of AAVhum.8 sc-puroguide-eGFP virus representing a theoretical MOI of 50,000. This high MOI was used to ensure that every cell in the library is infected, and infected cells were marked by eGFP expression. At 3 days postinfection, the cells were treated with 3 μg/ml puromycin for a week with medium changes every 2 to 3 days. Once >80% cell death was achieved, another round of infection with AAVhum.8 sc-puroguide-eGFP was carried out, followed by selection with puromycin for another week. This allowed robust selection and expansion of hum.8-resistant cell populations. Similar sets of uninfected library of cells were harvested 4 days after the initial plating and stored as cell pellet at −20°C. Genomic DNA was harvested from infected/selected and uninfected library cell pellets, sgRNA was amplified by PCR and subjected to NGS using MiSeq, as previously described (51).

### Generation of ITGB1 and AAVR knockout cell lines.

A previously validated sgRNA targeting the exon 3 of ITGB1 cloned into a Cas9-expressing lentivector was obtained as a gift from Stanley Lemon (41). Lentivirus was generated as described previously (52), transduced on Huh7 cells for 24 h, and selected with 3 μg/ml puromycin for a week until individual colonies of cells were formed. To generate the AAVR KO cell line, a previously validated sgRNA targeting exon 3 of AAVR was cloned into lenticrisprV2 plasmid, and lentivirus was generated as previously described (50). Huh7 cells were transduced for 24 h and selected with 10 μg/ml blasticidin for a week until individual colonies of cells were formed. Colonies were trypsinized and either used for experiments or frozen down. Both ITGB1 and AAVR knockouts were confirmed by Western blotting. Difficulty was encountered in attempting to isolate single ITGB1 knockout clones, so a polyclonal line was used as it still exhibited a robust reduction in protein and a clear transduction phenotype. Sanger sequencing of the sgRNA target region revealed a frameshift caused by a single nucleotide insertion.

### Cryo-EM data collection.

Quantifoil grids with a thin carbon film over the holes were glow discharged in a Pelco easiGlow (Ted Pella, Inc., Redding, CA) for 30 s prior to sample loading. Small volumes of purified AAVhum.8 vectors (∼3 μl/grid) were loaded onto the prepared grids and vitrified in liquid ethane by a Vitrobot Mark IV (FEI, Hillsboro, OR) freezing robot and subsequently transferred into liquid nitrogen. The capsid distribution and ice quality of the grids were examined using a 16-megapixel CCD camera (Gatan, Inc.) in a Tecnai (FEI Co.) G2 F20-TWIN transmission electron microscope operated at a voltage of 200 kV using low-dose conditions (∼20 e/Å^2^). Optimal grids were used for collecting micrograph movie frames using the Leginon semiautomated application (53) on a Titan Krios electron microscope (FEI Co.) operated at 300 kV at a total dose of 60 e^−^/Å^2^ recorded on a Gatan K3 direct electron detection camera with 50 movie frames per micrograph. The data were collected at the Biological Science Imaging Resource of the Florida State University as part of the NIH Southeastern Center for Microscopy of MacroMolecular Machines (SECM4) project.

### Cryo-EM image reconstructions.

The movie frames were aligned using MotionCor2 with dose weighting (54). For 3D-image reconstruction, the cisTEM software package was utilized (55). Briefly, the aligned micrographs were imported into the application, and their CTF parameters were estimated. This information was used to eliminate micrographs of poor quality. This was followed by automatic particle picking using a particle radius of ∼130 Å. This set of particles was subjected to 2D classification to eliminate non-AAV particles (ice and debris) from the automatic picking process. After the 2D classification, the capsid-complex particles were reconstructed using default settings. This included the *ab initio* 3D-model generation, autorefinement, and density map sharpening with a precutoff B-factor value of −90 Å^2^ and variable postcutoff B-factor values such as 0, 20, and 50 Å^2^. The sharpened density maps were inspected in the Coot and Chimera applications (56, 57). The −90 Å^2^/0 Å^2^ sharpened maps were used for assignment of the amino acid main and side chains. The resolutions of the cryoreconstructed density maps were estimated based on a Fourier shell correlation of 0.143.

### Model building, structure refinement, and structural comparison.

The AAVhum.8 VP3 atomic model was generated using the deposited capsid structure of AAV8 (PDB ID 6V12) as a template and the AAVhum.8 VP3 amino acid sequence in Swiss Model (58). A 60mer version of the model was generated using the ViperDB server (59) that was subsequently oriented and positioned into the cryo-EM map using the “Fit in Map” option in Chimera while maximizing the correlation coefficient (CC). The EMAN2 subroutine e2proc3d.py was implemented to resize the map based on the best fit as determined by CC from Chimera (56, 60). The AAVhum.8 VP model was manually real-space refined in Coot (57) by adjusting the Cα-backbone and amino acid side chains into the density of the cryo-EM map. A 60-mer of the VP3 model was further refined against the map with Phenix (61). Final statistics for the AAVhum.8 coordinates are provided in Table 1. Visual representations of maps and models were generated using UCSF Chimera (56). The overall paired root mean square deviations (RMSDs) between Cα positions to the AAV8 capsid structure was obtained in Coot by superposing the VP models.

### Statistical analysis.

Statistical analysis was performed with the GraphPad Prism software by one-way or two-way analysis of variance (ANOVA). *P* values of <0.05 are considered significant and denoted in the figures by asterisks (*, *P < *0.05; **, *P < *0.01; ***, *P < *0.001; ****, *P < *0.0001). Error bars represent standard deviations (SD) from the mean.

### Data availability.

The AAVhum.8 cryo-EM reconstructed density map and model built for the capsids were deposited in the Electron Microscopy Data Bank (EMDB) with the accession numbers EMD-23516 and PDB ID 7LTM, respectively.
